# Clinical trial on the effects of oral magnesium supplementation in stable-phase COPD patients

**DOI:** 10.1007/s40520-021-01921-z

**Published:** 2021-07-14

**Authors:** Bruno Micael Zanforlini, Chiara Ceolin, Caterina Trevisan, Agnese Alessi, Daniele Michele Seccia, Marianna Noale, Stefania Maggi, Gabriella Guarnieri, Andrea Vianello, Giuseppe Sergi

**Affiliations:** 1 Department of Medicine (DIMED), Geriatrics Division, University of Padua, Via Giustiniani 2, 35128 Padua, Italy; 2 National Research Council, Neuroscience Institute, Padua, Italy; 3 Respiratory Pathophysiology Unit, Department of Cardiological, Thoracic and Vascular Sciences, University of Padua, Padua, Italy

**Keywords:** Chronic obstructive pulmonary disease, Clinical trial, Magnesium, Inflammation

## Abstract

**Background and aims:**

COPD is a common chronic condition in older age that impacts on daily activities and quality of life. Previous studies suggest that magnesium deficit in COPD patients affects bronco-obstruction, inflammation, and physical performance. We investigated whether oral magnesium supplementation in stable-phase COPD patients improves lung function, physical performance, and quality of life.

**Methods:**

We conducted a double-blind randomized-controlled clinical study with 49 participants divided into two groups: one given 300 mg/day of magnesium citrate (*n* = 25) and the other one sachet/day of a placebo (*n* = 24). The following parameters were assessed at baseline and after 3 and 6 months: lung function (spirometry), physical performance (handgrip strength, lower limb strength, six-minute walk test), inflammation (e.g., C-reactive protein, CRP), disease-related symptoms, and quality of life (St George’s Respiratory Questionnaire, EuroQoL-5D, the Modified British Medical Research Council Questionnaire).

**Results:**

Linear mixed models revealed significantly lower CRP values in the intervention group than in the placebo group at the 6 month follow-up (*β* = − 3.2, 95% CI − 6.0, − 0.4, *p* = 0.03). Moreover, the maximum work for flexion tended to increase in both groups between the 3 and the 6 month assessments, especially in the placebo group. No significant differences within and between groups over the study period were observed for the other parameters tested.

**Conclusions:**

Although the established minimum sample size was not reached, our results suggests that oral magnesium supplementation may have a potential anti-inflammatory role. On the other hand, it does not seem to substantially influence lung function, physical performance, and quality of life in COPD patients.

**Trial registration:**

The study is registered in clinicaltrial.gov (Trial Registration: NCT02680769, 13 June 2016, retrospectively registered).

**Supplementary Information:**

The online version contains supplementary material available at 10.1007/s40520-021-01921-z.

## Introduction

The prevalence of chronic obstructive pulmonary disease (COPD) increases with age, reaching 14.2% in the over 65 s making it a common chronic disorder of old age [1]. This also explains the frequent co-existence of COPD with other chronic conditions, such as cardiovascular and musculoskeletal disorders, or diabetes mellitus, which can heavily impact a patient’s quality of life; for this reason, for assessing the severity of the disease the GOLD guidelines also take into account the impact of symptoms on daily activities [2].

Several studies have shown that the total magnesium pool tends to decrease both in COPD and in the elderly [3, 4]. This deficiency is often overlooked, because it is not easily identifiable by the simple dosage of serum magnesium [5]. Magnesium may antagonize calcium, blocking its channels and hindering the release of acetylcholine and its action on the neuromuscular plate [6–8]. It therefore plays an important role in muscle strength and exercise performance [9]. Magnesium deficiency could also trigger a low-grade inflammatory state [4] through increased activation of neutrophils and release of histamine from mast cells. In COPD, these effects would lead to acceleration of the pathophysiological mechanisms of the disease [3, 5], maintenance of a condition of chronic sub-inflammation, and an increase in cellular senescence [5]. Magnesium deficiency could therefore result in a higher frequency of exacerbations [1, 5], an increase in bronchoconstriction, and a reduction in physical performance [9].

Previous studies have investigated the effects of magnesium supplementation in COPD patients. The results suggest that it may reduce lung hyperinflation, and increase the strength of the respiratory muscles [10] and the bronco-dilating action of beta-2 agonists, with subsequent improvement in peak expiratory flow [11]. In most of these studies, supplementation was in the form of intravenous magnesium sulfate, while only a few investigated the impact of inhaled magnesium on FEV1 in the course of COPD exacerbation, without finding any benefit [12, 13]. Ours is the first clinical trial investigating the effect of oral administration of magnesium on lung function, physical performance, and quality of life in clinically stable COPD.

## Materials and methods

### Study population

The study was carried out with patients recruited at the Respiratory Physiopathology Unit of the University Hospital of Padua (Italy), which they attended for periodic control visits for COPD. Recruitment was carried out from March 2016 to December 2017.

The inclusion criteria were: age over 18; moderate–severe COPD (FEV1 30–80% of the predicted value); ability to perform spirometry, strength and physical performance tests; body mass index (BMI) between 18.6 and 34.9 kg/m^2^. The exclusion criteria were: recent hospitalization for respiratory problems (in the 30 days prior to screening); ongoing treatment with theophylline, insulin, and/or steroids (with a dosage greater than 5 mg prednisone equivalent); active cancer (positive screening in the last 5 years); severe kidney disease (GFR < 60 ml/min); chronic liver disease (transaminases greater than twice the upper limit of normal); oral supplementation with magnesium or calcium.

The study design was authorized by the local Ethics Committee (Comitato Etico per la sperimentazione clinica della provincia di Padova) and respected the guidelines of the Declaration of Helsinki. Each participant gave written informed consent to participate in the study.

### Study design and intervention

Pulmonologists and geriatricians recruited and selected patients according to the above-mentioned inclusion criteria. Thirty-eight packs containing magnesium citrate (300 mg/pack) and 38 packs containing the placebo were prepared (76 packs; the expected number of participants). The placebo contained the same ingredients as the verum without magnesium citrate (substituted with a higher amount of maltodextrin to reach the same total amount of 5 g per sachet), namely: maltodextrin, riboflavin (Vitamin B2), orange flavor, citric acid, sucrose, and sodium bicarbonate. Neither the packs nor the sachets had any identification mark that could distinguish the placebo and the verum product. The participant’s numbers from 1 to 76 were randomly assigned by the Protina Pharmazeutische GmbH (that provided the magnesium and placebo pack) using a computer random number generator (www.random.org) to the magnesium (intervention) or the placebo (control) group, and then applied to the packs accordingly. Upon recruitment, each patient was assigned a pack in order from 1 to 76: neither the investigator nor the patient was aware of the pack’s contents. The pack number assigned to the participant was reported in his/her case report form (CRF) to ensure traceability of the information and the anonymity of the participant. Once the recruited patients had been allocated to the intervention or control groups, they underwent a baseline assessment, with further assessments after 3 months (first follow-up) and after 6 months (second follow-up). Every month research staff contacted the participants by telephone to inquire as to their progress, and whether there had been any reason to interrupt the intake or any adverse effects. The study is registered in clinicaltrial.gov (Trial Registration: NCT02680769).

### Participant assessments

#### Anthropometry

Body weight and height were measured with participants wearing light indoor clothing and without shoes. BMI was calculated as body weight in kilograms divided by height in meters squared.

#### Laboratory data

Venous blood samples were analyzed for the following biochemical parameters: C-reactive protein (CRP), serum magnesium, and tumor necrosis factor-α (TNF-α). At the recruitment phase only, we also measured the levels of alanine transaminase (ALT), aspartate transaminase (AST), and creatinine to detect the presence of kidney or liver dysfunction. The analyses were performed following standard procedures at the laboratory unit of the University Hospital of Padua, which has Clinical Pathology Accreditation.

#### Spirometry

Spirometry was performed with a Spirometer Pony FX (Cosmed Ltd., Italy) calibrated according to the manufacturer’s technical instructions and administered by the Respiratory Physiopathology Unit team. The values obtained were: forced expiratory volume in one second (FEV1), forced expiratory volume in six seconds (FEV6), forced vital capacity (FVC), Tiffeneau index (FEV1/FVC), peak expiratory flow (PEF), maximum expiratory flow at 25% of FVC (MEF25%), maximum expiratory flow at 50% of FVC (MEF50%), maximum expiratory flow at 75% of FVC (MEF75%), forced expiratory flow between 25 and 75% of FVC (FEF25-75%), forced expiration time at 100% of FVC (FET100%), and retrograde extrapolation volume (VEXT). The parameters measured were compared with ​​expected normal values according to the European Respiratory Society (ERS-93).

#### Physical performance

Lower limb strength was assessed by isometric tests against a fixed resistance, and isotonic tests against mobile resistance. The evaluation was carried out with a PrimaDOC isokinetic dynamometer (Easytech, Italy). The following parameters were measured: maximum flexion moment, maximum extension moment, maximum flexion strength, maximum extension strength, maximum flexion power, maximum extension power, maximum isometric moment, and isometric strength. Upper limb strength was evaluated through three repetitions of the handgrip strength test (maximum handgrip strength) and one repetition of the handgrip endurance test (maximum handgrip endurance). Measurements were made with DynEx electronic hand dynamometers (Ohio, USA) by trained personnel. Exercise tolerance was assessed with the 6-minute walk test (6MWT): patients were asked to walk at their usual pace up and down a 30-m corridor, and the distance covered in 6 min was recorded.

#### COPD symptoms and impact on quality of life

Participants self-administered the following questionnaires:

- Modified British Medical Research Council (mMRC) Questionnaire, which measures the degree of dyspnea. An mMRC score ≥ 2 indicates a patient with significant symptoms.
- St George’s Respiratory Questionnaire (SGRQ), which investigates symptoms and quality of life in COPD and has three sections: symptoms, activities, and impact. The overall score ranges from 0 (no impairment) to 100 (maximum impairment). Scores ≥ 25 are very rare in healthy people.
- EuroQoL-5D, which assesses quality of life in terms of mobility, personal care, daily activities, pain or discomfort, and anxiety and depression. The interviewee expresses a judgment on the perceived impact of each dimension on their life on a scale of 0–3.

### Statistical analysis

The sample size was calculated on the difference between the 6 month and baseline assessments in the primary outcome variable, i.e., FEV1, the distribution of which had been approximated to normal (for a sufficiently high number) with a standard deviation (SD) of 150 ml. Assuming a power of 80% and a significance level of 5% in the two-tailed test, we arrived at 32 as the estimated number of people per group sufficient to evidence a statistically significant variation of 100 ml, if actually present. We chose the cut-off of 100 ml, since it was previously proposed as minimal clinically important difference for COPD patients in intervention studies [14, 15]. With an expected drop-out rate of 20%, the final number was estimated at 76 people overall (38 per group). For the secondary outcomes, assuming a power of 80% 76 was the number of people estimated as being sufficient to evidence a statistically significant difference of 4 points (SD = 2) in the SGQR scores, and of 103 m (SD = 140 m) in the 6MWT. This number was also estimated to be able to evidence a statistically significant difference in the flexion and extension strength of the tibial segment of 1 kg (SD = 2.5 kg), and in hand strength (handgrip) of 1 kg (SD = 2.5 kg).

The participant’s characteristics were expressed as mean ± SD for normally distributed quantitative variables, medians (25th–75th percentile) for non-normally distributed quantitative variables, and as counts and percentages for categorical variables. The characteristics of the intervention and control groups were compared with the Student’s *t* test for independent samples for parametric variables, the Kolmogorov–Smirnov test for non-parametric variables, and the Chi-square or Fisher’s test for categorical variables. Longitudinal analysis of the data (baseline, 3- and 6-month follow-ups) for the primary and secondary outcomes was performed with linear mixed models adjusted for significantly different variables in the two groups at baseline (SGRQ total score). First, we evaluated whether the differences between the intervention and control groups changed at each follow-up by testing the group^*^time interaction. Second, we evaluated the changes within each group at 3 and 6 months from baseline with the Tukey–Kramer adjustment for multiple comparisons. Estimates were expressed as beta coefficients (*β*) with 95% confidence intervals (95% CI). An intention-to-treat analytical method was adopted, i.e., individuals initially enrolled in the study contributed to the analyses until their last observation. Analyses were performed in IBM SPSS version 25 (IBM Corp., Armonk, NY) and in SAS 9.4 (SAS Institute Inc., Cary, NC).

## Results

Of a total of 198 COPD patients screened, 49 agreed to participate in the study and were randomized into the placebo (*n* = 24) or the intervention (*n* = 25) group (for the study flowchart with exclusions and drop-outs, see Supplementary Fig. 1). Because of difficulties in the participant’s enrollment, the study did not reach the sample size calculated a priori. Each participant was provided with a pack of 180 sachets of either the supplement or the placebo and instructed to take the contents of one sachet every day for 180 days. Participants were instructed not to take more than one sachet on a given day if a dose had been missed, and to report the number of missed doses at the monthly telephone interview.

The characteristics of the sample at baseline and the results of the serological tests, spirometry, physical performance tests, and questionnaires are shown in Table 1. As can be seen, there were no significant differences between the groups, except for the SGRQ total and daily activities scores, which were higher in the placebo group. Only one patient had serum magnesium just below the expected range of normality**.**

**Table 1:** Characteristics of the sample at baseline

	All (*n* = 49)	Magnesium (*n* = 25)	Placebo (*n* = 24)	*p* value
Age (years)	72.6 ± 9.9	73.0 ± 8.9	72.2 ± 11.0	0.77
Sex—female	11 (22.4%)	6 (24.0%)	5 (20.8%)	0.79
BMI (kg/m^2^)	27.0 ± 4.2	26.9 ± 4.3	27.1 ± 4.3	0.87
Number of drugs	5 (4.0–7.5)	6 (4.0–7.5)	5 (3.0–7.5)	0.46
Comorbidities				
Arthrosis	5 (10.2%)	3 (12.0%)	2 (8.3%)	1.00
Cardiovascular diseases	19 (38.8%)	13 (52.0%)	6 (25%)	0.05
Hypertension	25 (51.1%)	16 (60%)	10 (41.7%)	0.26
Current smoking habits	10 (20.4%)	5 (20.0%)	5 (20.8%)	0.58
GOLD classification				
A	19 (38.8%)	11 (44.0%)	8 (33.3%)	0.34
B	6 (12.2%)	1 (4.0%)	5 (20.8%)	
C	16 (32.7%)	9 (36.0%)	7 (29.2%)	
D	8 (16.3%)	4 (16.0%)	4 (16.7%)	
Serological tests				
Magnesium (nmol/L]	0.8 ± 0.1	0.8 ± 0.1	0.8 ± 0.1	0.65
TNF-α (ng/L)	6.1 (5.3–7.8)	6.1 (5.3–7.2)	6.7 (5.3–8.2)	0.34
CRP (mg/L)	1.5 (1.5–6.3)	1.5 (1.5–5.4)	1.5 (1.5–6.7)	0.67
Spirometric parameters				
FEV1 (%)	1.4 ± 0.5	1.4 ± 0.6	1.4 ± 0.6	0.81
FEV1/FVC (%)	56.3 ± 11.2	56.3 ± 13.1	56.3 ± 9.2	0.99
Physical performance tests				
Max handgrip (kg)	32.2 ± 9.0	33.1 ± 10.0	31.3 ± 8.1	0.49
6MWT (m)	408.0 (350.0–449.0)	412.0 (351.0–452.0)	404.0 (323.0–424.0)	0.54
Flex peak torque (Nm)	28.8 ± 10.9	28.2 ± 9.4	29.5 ± 12.4	0.68
Ext peak torque (Nm)	67.0 ± 20.1	66.7 ± 18.9	67.4 ± 21.7	0.90
Isom M max (Nm)	98.3 ± 29.2	95.6 ± 31.2	101.0 ± 27.2	0.52
Questionnaires				
SGRQ total	27.0 ± 14.4	21.7 ± 11.7	32.5 ± 15.2	0.01
SGRQ activities	41.6 ± 22.6	34.5 ± 22.8	49.3 ± 20.3	0.03
SGRQ impact	17.4 (9.8–28.5)	15.7 (6.0–23.5)	18.8 (11.2–33.4)	0.12
SGRQ symptoms	22.9 (13.2–37.4)	17.8 (11.0–31.0)	26.2 (17.9–42.9)	0.08
EQ5D	0.9 (0.7–1.0)	0.9 (0.8–1.0)	0.9 (0.7–1.0)	0.54
EQ5D VAS	70.0 (60.0–80.0)	70.0 (61.3–80.0)	67.5 (50.0–80.0)	0.35
MRC	1.0 (0.0–2.0)	1.0 (0.0–1.3)	1.0 (0.5–2.0)	0.55

Numbers are mean ± SD, median (interquartile range), or count (%), as appropriate

*BMI* body mass index, *TNF-α* tumor necrosis factor-α, *CRP* C-reactive protein, *FEV1* forced expiratory volume in one second, *FEV1/FVC* Tiffeneau index, *PEF* peak expiratory flow, *Max handgrip* maximum handgrip strength, *6MWT* six-minute walk test, *Flex peak torque* maximum flexion moment, *Ext peak torque* maximum extension moment, *Isom* *M* *max* maximum isometric moment, *SGRQ* St George’s Respiratory Questionnaire, *EQ5D* EuroQoL-5D, *VAS* visual analogue scale, *MRC* Modified British Medical Research Council Questionnaire

**Fig. 1:**
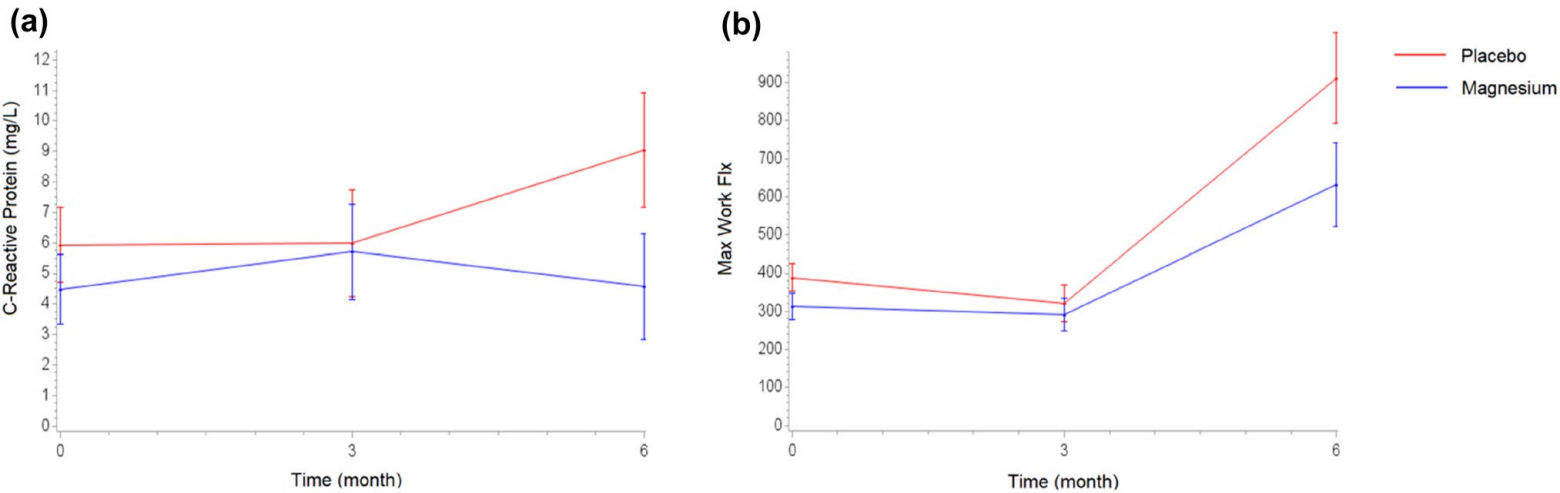
Estimated means (standard error) of C-reactive protein (**a**) and maximum flexion strength over time, by treatments

Table 2 and Supplementary Table 1 show the results from the linear mixed models for the outcomes evaluated. The only significant differences between the intervention and the placebo groups over time were in the CRP values and maximum flexion strength. As Fig. 1a shows, at the 6-month follow-up the intervention group had significantly lower CRP values than the placebo. Supplementary Tables 2 and 3 suggest that this difference was determined by a significant increase in CRP values in the placebo group, and not by a decrease in the intervention group; the size of the effect of this difference, considering our final sample size, was 0.40 [16].

**Table 2:** Differences between the Mg and placebo groups at each study assessment estimated by linear mixed models

Outcome	Beta coefficients (95% confidence intervals)		
	Group (Mg vs placebo)	Group^*^time (3 months)	Group^*^time (6 months)
Magnesium	0.0 ( − 0.0, 0.1) *p* = 0.46	0.0 ( − 0.0, 0.1) *p* = 0.42	0.0 ( − 0.0, 0.1) *p* = 0.78
TNF-α	−1.0 ( − 2.6, 0.7) *p* = 0.23	0.2 ( − 1.7, 2.0) *p* = 0.85	0.5 ( − 1.6, 2.7) *p* = 0.61
C-reactive protein	− 1.5 ( − 5.2, 2.2) *p* = 0.43	1.0 ( − 2.0, 4.1) *p* = 0.48	− 3.2 ( − 6.0, − 0.4) *p* = 0.03
FEV1	− 0.1 ( − 0.2, − 0.0) *p* = 0.03	0.0 ( − 0.04, 0.04) *p* = 0.98	− 0.02 ( − 0.1, 0.03) *p* = 0.46
FEV1/FVC	− 4.2 ( − 11.0, 2.5) *p* = 0.21	0.7 ( − 2.9, 4.2) *p* = 0.71	− 1.0 ( − 3.8, 1.8) *p* = 0.47
PEF	− 1.0 ( − 1.9, − 0.1) *p* = 0.03	0.3 ( − 0.5, 1.0) *p* = 0.44	0.0 ( − 0.4, 0.5) *p* = 0.85
Max handgrip	2.1 ( − 3.9, 8.1) *p* = 0.49	− 1.9 ( − 4.4, 0.6) *p* = 0.13	− 1.1 ( − 3.4, 1.2) *p* = 0.34
6MWT	− 41.8 ( − 88.9, 5.4) *p* = 0.08	− 2.8 ( − 45.2, 39.7) *p* = 0.90	24.8 ( − 29.0, 78.7) *p* = 0.35
Flex peak torque	− 4.3 ( − 10.9, 2.3) *p* = 0.20	3.9 ( − 3.4, 11.3) *p* = 0.29	− 1.7 ( − 8.1, 4.9) *p* = 0.61
Ext peak torque	− 7.0 ( − 19.6, 5.6) *p* = 0.27	3.8 ( − 5.9, 13.4) *p* = 0.43	3.4 ( − 5.5, 12.2) *p* = 0.45
Isom M max	− 5.7 ( − 22.6, 11.2) *p* = 0.50	− 1.3 ( − 15.2, 12.6) *p* = 0.85	7.8 ( − 5.0, 20.7) *p* = 0.23
SGRQ total	− 11.1 ( − 19.0, − 3.2) *p* = 0.01	2.2 ( − 4.5, 8.9) *p* = 0.52	0.3 ( − 5.8, 6.4) *p* = 0.91
SGRQ activities	− 15.9 ( − 28.4, − 3.5) *p* = 0.01	3.8 ( − 8.8, 16.5) *p* = 0.55	7.8 ( − 2.7, 18.4) *p* = 0.14
SGRQ impact	− 7.3 ( − 15.5, 0.9) *p* = 0.08	0.6 ( − 6.1, 7.2) *p* = 0.87	− 2.7 ( − 9.3, 3.9) *p* = 0.42
SGRQ symptoms	− 7.7 ( − 16.9, 1.6) *p* = 0.10	1.7 ( − 9.6, 13.1) *p* = 0.76	− 7.5 ( − 19.0, 4.0) *p* = 0.20
EQ5D	0.0 ( − 0.1, 1.8) *p* = 0.53	− 0.0 ( − 0.1, 0.6) *p* = 0.52	0.0 ( − 0.1, 0.1) *p* = 0.90
EQ5D VAS	7.0 ( − 2.8, 16.9) *p* = 0.16	− 2.9 ( − 11.5, 5.8) *p* = 0.51	3.8 ( − 5.0, 12.6) *p* = 0.39
MRC	− 0.1 ( − 0.6, 0.3) *p* = 0.56	− 0.1 ( − 0.5, 0.4) *p* = 0.72	− 0.3 ( − 0.9, 0.3) *p* = 0.36

Coefficients are derived from linear mixed models including group, time, and group^*^time, adjusted for SGRQ total (except when SGRQ scales are the outcomes); the intercept is set as random

*TNF-α* tumor necrosis factor-α, *FEV1* predicted FEV1 (forced expiratory volume in one second), *FEV1/FVC* Tiffeneau index, *PEF* peak expiratory flow, *Max handgrip* maximum handgrip strength, *6MWT* six-minute walk test, *Flex peak torque* maximum flexion moment, *Ext peak torque* maximum extension moment, *Isom M max* maximum isometric moment, *EQ5D* EuroQoL-5D, *VAS* visual analogue scale, *MRC* Modified British Medical Research Council Questionnaire, *SGRQ* St George’s Respiratory Questionnaire

The trend in maximum flexion strength (Fig. 1b) suggests that both groups tended to increase their performance between the 3- and 6-month assessments, especially the placebo group (Supplementary Tables 2 and 3).

### Discussion and conclusions

This is the first double-blind, randomized-controlled clinical trial investigating the effect of oral administration of magnesium on lung function, physical performance, and quality of life in clinically stable COPD patients.

Previous studies suggest that hypomagnesaemia may affect the hyperactivity of the airways. Indeed, magnesium normally relaxes bronchial smooth muscle by blocking calcium-dependent channels, and inhibits the release of acetylcholine from neuromuscular junctions [17]. The mechanisms through which magnesium levels may influence respiratory performance in COPD patients are still unclear, and those studies that have investigated the effects of magnesium supplementation on lung function have reported conflicting results.

In our study, we found no significant changes in respiratory parameters as a result of magnesium supplementation, which we acknowledge could have been due to the failure to reach the predetermined sample size. However, Fogarty and colleagues also found oral administration of magnesium to have no effect on lung function in a population of asthmatic patients [18]. As in the work of Fogarty et al., magnesium levels in our sample were on average normal at baseline. Similar results were reported for intravenous administration of magnesium, which was found not to influence FEV1 in the stable phase of COPD [10]. The results regarding post-bronchodilator FEV1 are conflicting, since some studies found significant improvement in the magnesium group compared with the placebo group [17, 19, 20], while others found no significant change [10–13, 21, 22]. Although these studies are highly varied, it should be borne in mind that an improvement in FEV1 in response to β2-agonist has been often associated with intravenous magnesium administration. This suggests that magnesium may improve the response of the bronchial musculature to a bronchodilator through its anti-inflammatory properties and its role in the regulation of muscle contraction [6, 8, 23, 24], which could be more important during exacerbation.

The analysis of biochemical parameters revealed increased CRP values in the placebo group. This suggests that oral magnesium supplementation may have an anti-inflammatory role. Some studies investigating magnesium supplementation through other means of administration have obtained similar results [25, 26]. However, Kazaks and colleagues, who analyzed the effects of oral magnesium supplementation in a population of asthmatic subjects, reported no significant changes in inflammation markers in either the placebo or the magnesium group [27].

Interestingly, we did not observe any significant changes in serum magnesium levels in the intervention group compared with the placebo group. Some studies analyzing patients without respiratory diseases found an increase in serum magnesium levels within a few days or weeks of oral magnesium supplementation [28, 29]. A possible explanation for the differences with our findings may lie in the fact that administration through sachets and non-effervescent preparations reduces the amount of ionized magnesium in the circulation [30], which represents about 60–70% of the circulating magnesium and therefore its active part. [29] On the other hand, the evaluation of serum magnesium may not represent the better index of body storage, because extracellular magnesium represents only 1% of the total amount of body and seems to be strictly regulated. In this regard, the assessment of urinary magnesium concentrations might better quantify the variation of this ion in the body [31], although we had not the possibility to test it.

Regarding physical performance, we found a trend towards improvement in lower limb strength expressed as maximum flexion strength in both groups. This could be linked to the participant’s greater commitment to exercise during the study period, or a placebo effect of being involved in a clinical trial, even though patients did not know whether they were assigned to the magnesium or placebo groups. Do Amaral et al. [32] investigated the effect of intravenous administration of magnesium on physical performance in stable COPD and found an increase in maximal exercise capacity performed on a cycle ergometer. However, it is not possible to compare this study with ours as they differ in the means of magnesium administration and the methods used to assess physical performance.

Finally, we did not obtain any significant results regarding dyspnea symptoms and quality of life over the follow-up period in either of the groups. Up to now, a few clinical studies have investigated the effects of magnesium on quality of life and overall symptoms. Those studies that measured patient’s dyspnea levels using visual-spatial or analog scales found that magnesium administration was not associated with any substantial differences in dyspnea scores [21, 22], in line with our findings.

The major limitation of the present study is that we did not reach the sample size calculated a priori as being necessary to detect possible significant effects of the intervention on the primary outcome. The observed variations, although statistically significant, should be investigated in greater depth to determine whether they translate into clinically significant changes in COPD patients. On the other hand, the strengths of the study lie in its design and the large set of data collected from each participant.

In conclusion, this is the first double-blind randomized-controlled clinical trial investigating the effects of orally administered magnesium on lung function, physical performance, and quality of life in people with stable COPD. The results support a possible anti-inflammatory role of orally administered magnesium in this category of patients. However, there is a need for further investigation with a larger sample to explore the benefits of magnesium on COPD patients at different stages of the disease and with respect to different outcomes.

## Supplementary Information

Below is the link to the electronic supplementary material.

Supplementary file1 (DOCX 28 KB)

Supplementary file2 (DOCX 40 KB)

## Data Availability

Not applicable.
