# Supplementary material for: Clinical trial on the effects of oral magnesium supplementation in stable-phase COPD patients
Source: Aging Clin Exp Res. 2021 Jul 14;34(1):167–74. doi: 10.1007/s40520-021-01921-z (PMC8794984; doi:10.1007/s40520-021-01921-z)
Supplement: Supplementary file 1 — Supplementary file1 (DOCX 28 KB) [file 40520_2021_1921_MOESM1_ESM.docx]

**Supplementary Table 1. Estimated differences between Mg and placebo groups at each study assessment**

| **Outcome** | **Beta coefficients (95% Confidence Intervals)** | | |
| --- | --- | --- | --- |
|  | **Group (Mg vs placebo)** | **Group*time**  **(3 month)** | **Group*time**  **(6 month)** |
| **FVC** | -0.4 (2.7, 3.8)  p=0.09 | -0.0 (-0.3, 1.4)  p=0.54 | 1. (-0.1, 0.2)   p=0.73 |
| **FET100%** | -0.3 (-1.0, 0.5)  p=0.47 | 0.3 (-0.5, 1.1)  p=0.49 | -0.1 (-0.8, 0.6)  p=0.77 |
| **Max endurance** | 2.4 (-25.1, 29.9)  p=0.86 | -0.2 (-29.7, 29.4)  p=0.99 | -2.8 (-27.5, 21.8)  p=0.82 |
| **Max flx strength** | -75.8 (-180.2, 28.5)  p=0.15 | 46.8 (-58.1, 151.8)  p=0.37 | -204.4 (-492.2, 83.3)  p=0.16 |
| **Max ext strength** | -75.2 (-252.4, 101.9)  p=0.40 | -44.0 (-161.0, 72.9)  p=0.45 | -29.9 (-128.8, 68.9)  p=0.54 |
| **Max flx power** | -89.8 (-204.1, 24.5)  p=0.12 | 87.7 (-37.5, 212.9)  p=0.16 | -21.3 (-141.6, 99.1)  p=0.72 |
| **Max ext power** | -110.8 (-322.4, 100.8)  p=0.30 | 49.5 (-109.8, 208.7)  p=0.53 | 44.0 (-105.8, 193.9)  p=0.55 |
| **Isom M max** | -5.7 (-22.6, 11.2)  p=0.50 | -1.3 (-15.2, 12.6)  p=0.85 | 7.8 (-5.0, 20.7)  p=0.23 |

Coefficients are derived from linear mixed models including group, time, and group*time, adjusted for SGRQ total (except when SGRQ scales are the outcomes); the intercept is set as random. Abbreviations: FVC = forced vital capacity; FET100% = forced expiration time at 100% of FVC; Max endurance = maximum handgrip endurance; Max flx strength = maximum flexion strength; Max ext strength = maximum extension strength; Max flx power = maximum flexion power; Max ext power = maximum extension power; Isom M max = maximum isometric moment.

**Supplementary Table 2. Estimated within-group T0, T3, and T6 values, and within-group mean differences (T3-T0 and T6-T0) in the magnesium group**

|  | **Magnesium** | | | | | | |
| --- | --- | --- | --- | --- | --- | --- | --- |
|  | **T0**  **Mean (SE)** | **T3**  **Mean (SE)** | **T6**  **Mean (SE)** | **Mean**  **Difference**  **T3 – T0 (SE)** | **p-**  **value^§^** | **Mean**  **Difference**  **T6 – T0 (SE)** | **p-**  **value^§^** |
| FEV1/FVC | 54.4 (2.2) | 56.2 (2.4) | 54.2 (2.2) | 1.85 (1.10) | 0.56 | -0.18 (0.93) | 1.00 |
| FEV1% | 0.56 (0.03) | 0.58 (0.04) | 0.54 (0.04) | 0.02 (0.01) | 0.78 | -0.02 (0.02) | 0.95 |
| FVC | 2.37 (0.15) | 2.31 (0.16) | 2.31 (0.16) | -0.05 (0.07) | 0.96 | -0.05 (0.06) | 0.94 |
| FEV6 | 2.33 (0.14) | 2.33 (0.16) | 2.28 (0.17) | 0.01 (0.06) | 0.96 | -0.05 (0.06) | 0.98 |
| PEF | 3.26 (0.3) | 3.17 (0.34) | 3.19 (0.32) | -0.09 (0.23) | 1.00 | -0.04 (0.06) | 1.00 |
| FET100% | 6.57 (0.25) | 6.08 (0.26) | 6.15 (0.15) | -0.50 (0.26) | 0.42 | -0.42 (0.24) | 0.52 |
| Ext peak torque | 65.3 (4.2) | 66.4 (5) | 65.1 (4.6) | 1.01 (3.06) | 1.00 | -0.26 (3.00) | 1.00 |
| Flx peak torque | 27.3 (2.2) | 26.3 (3) | 24.8 (2.6) | -0.92 (2.34) | 1.00 | -2.46 (2.18) | 0.87 |
| Max ext power | 1030.2 (70) | 1024.5 (82.7) | 1016.5 (76.3) | -5.70 (50.6) | 1.00 | -13.7 (50.9) | 1.00 |
| Max flx power | 405.5 (37.9) | 404.3 (51) | 380.3 (46) | -1.26 (39.9) | 1.00 | -25.2 (40.2) | 0.99 |
| Max flx strength | 313.3 (34.9) | 292.4 (42.8) | 631.6 (110) | -20.9 (33.4) | 0.99 | 318.3 (96.3) | 0.03 |
| Max ext strength | 816.3 (58.8) | 784.5 (65.3) | 742.2 (57.3) | -31.8 (37.2) | 0.95 | -74.1 (33.8) | 0.27 |
| Isom M max | 96.4 (6.3) | 94.6 (6.4) | 101.6 (7.1) | -1.8 (4.6) | 1.00 | 5.1 (4.1) | 0.81 |
| Isom strength | 265.8 (17.1) | 257.1 (17.4) | 276.9 (19.3) | -8.7 (14.4) | 0.99 | 11.1 (13.2) | 0.96 |
| Max handgrip | 33.2 (2) | 32.5 (1.9) | 33.1 (1.9) | -0.8 (0.8) | 0.92 | -0.1 (0.8) | 1.00 |
| Max endurance | 87.2 (9.3) | 90 (8.9) | 87.7 (8.5) | 2.8 (9.6) | 1.00 | 0.5 (8.4) | 1.00 |
| 6MWT | 374.7 (15.7) | 362.9 (19.5) | 373.3 (19.6) | -11.7 (13.5) | 0.95 | -1.4 (18.4) | 1.00 |
| SGRQ symptoms | 24.6 (2.6) | 25.3 (3.4) | 23.4 (3.6) | 0.6 (3.9) | 1.00 | -1.2 (4.1) | 1.00 |
| SGRQ activity | 38.9 (2.3) | 38.6 (3.7) | 48.2 (3.4) | -0.3 (4.2) | 1.00 | 9.3 (3.7) | 0.15 |
| SGRQ impacts | 18.1 (1.2) | 16.8 (2.2) | 17.8 (2.5) | -1.3 (2.2) | 0.99 | -0.3 (2.3) | 1.00 |
| SGRQ total | 25.2 (1.5) | 24.4 (1.6) | 27.4 (1.7) | -0.8 (2.0) | 1.00 | 2.3 (2.1) | 0.89 |
| EQ5D VAS | 70.4 (2.9) | 69.1 (3) | 73.8 (3.2) | -1.6 (2.9) | 0.99 | 3.3 (3.2) | 0.90 |
| EQ5D tot | 0.85 (0.04) | 0.84 (0.03) | 0.86 (0.04) | -0.0 (0.03) | 1.00 | 0.0 (0.04) | 1.00 |
| MRC | 1.15 (0.13) | 1.07 (0.14) | 1.26 (0.21) | -0.1 (0.2) | 0.99 | 0.1 (0.2) | 1.00 |
| CRP | 3.0 (1.0) | 3.7 (1.0) | 3.2 (1.1) | 0.7 (0.6) | 0.86 | 0.2 (0.7) | 1.00 |
| Mg | 0.82 (0.02) | 0.84 (0.02) | 0.83 (0.02) | 0.02 (0.01) | 0.84 | 0.01 (0.02) | 0.99 |
| TNF | 5.3 (0.6) | 5.1 (0.6) | 5.5 (0.6) | -0.2 (0.4) | 0.99 | 0.2 (0.4) | 1.00 |

§: with Tukey-Kramer adjustment for multiple comparisons. Abbreviations: FEV1/FVC = Tiffeneau Index; FEV1% = predicted FEV1 (forced expiratory volume in one second); FVC = forced vital capacity; FEV6 = forced expiratory volume in six seconds; PEF = peak expiratory flow; FET100% = forced expiration time at 100% of FVC; Ext peak torque = maximum extension moment; Flx peak torque = maximum flexion moment; Max ext power = maximum extension power; Max flx power = maximum flexion power; Max ext strength = maximum extension strength; Max flx strength = maximum flexion strength; Isom M max = maximum isometric moment; Isom strength = isometric strength; Max handgrip = maximum handgrip strength; Max endurance = Maximum handgrip endurance; 6MWT = six-minute walk test; SGRQ = St George’s Respiratory Questionnaire; EQ5D = EuroQoL 5D; VAS = visual analogue scale; MRC = Modified British Medical Research Council Questionnaire; CRP = C-reactive protein; Mg = magnesium; TNF-α = tumor necrosis factor-α.

**Supplementary Table 3. Estimated within-group T0, T3, and T6 values, and within-group mean differences (T3-T0 and T6-T0) in the placebo group**

|  | **Placebo** | | | | | | |
| --- | --- | --- | --- | --- | --- | --- | --- |
|  | **T0**  **Mean (SE)** | **T3**  **Mean (SE)** | **T6**  **Mean (SE)** | **Mean**  **Difference**  **T3 – T0 (SE)** | **p-**  **value^§^** | **Mean**  **Difference**  **T6 – T0 (SE)** | **p-**  **value^§^** |
| FEV1/FVC | 58.6 (2.4) | 59.8 (2.7) | 59.4 (2.3) | 1.2 (1.4) | 0.95 | 0.8 (1.0) | 0.96 |
| FEV1% | 0.7(0.0) | 0.7 (0.0) | 0.7 (0.0) | 0.02 (0.02) | 0.88 | -0.00 (0.02) | 1.00 |
| FVC | 2.8 (0.2) | 2.8 (0.2) | 2.7 (0.2) | 0.01 (0.08) | 1.00 | -0.08 (0.06) | 0.76 |
| FEV6 | 2.6 (0.2) | 2.7 (0.2) | 2.6 (0.2) | 0.01 (0.08) | 1.00 | -0.08 (0.06) | 0.76 |
| PEF | 4.2 (0.3) | 3.9 (0.4) | 4.1 (0.3) | -0.4 (0.3) | 0.78 | -0.1 (0.2) | 0.98 |
| FET 100 | 6.8 (0.3) | 6.0 (0.3) | 6.5 (0.2) | -0.8 (0.3) | 0.15 | -0.3 (0.3) | 0.80 |
| Ext peak torque | 72.3 (4.4) | 69.6 (5.6) | 68.7 (4.9) | -2.7 (3.7) | 0.97 | -3.6 (3.2) | 0.86 |
| Flx peak torque | 31.6 (2.3) | 26.7 (3.5) | 30.7 (2.8) | -4.8 (2.8) | 0.52 | -0.8 (2.4) | 1.00 |
| Max ext power | 1141 (74.2) | 1085.8 (92.4) | 1083.3 (81.5) | -55.2 (60.7) | 0.94 | -57.7 (54.2) | 0.89 |
| Max flx power | 495.4 (40.1) | 406.4 (58.5) | 491.4 (50.5) | -89 (47.4) | 0.44 | -3.9 (44.2) | 1.00 |
| Max flx strength | 389.2 (36.7) | 321.4 (48.6) | 911.9 (119.5) | -67.8 (39.6) | 0.54 | 522.8 (105.7) | 0.00 |
| Max ext strength | 891.5 (62.2) | 903.7 (72.4) | 847.4 (60.9) | 12.2 (44.6) | 1.00 | -44.2 (35.6) | 0.81 |
| Isom M max | 103.5 (6.7) | 99.8 (7.2) | 98 (7.6) | -3.8 (5.4) | 0.98 | -5.6 (4.5) | 0.81 |
| Isom M mean | 91.9 (5.9) | 90.8 (6.5) | 87.8 (6.9) | -1.1 (5.3) | 1.00 | -4.1 (4.7) | 0.95 |
| Isom strength | 287.2 (18.1) | 276.1 (20) | 268.3 (20.9) | -11.1 (16.9) | 0.99 | -18.8 (14.4) | 0.78 |
| Max handgrip | 31.1 (2.1) | 32.3 (2.1) | 32.1 (2) | 1.1 (0.9) | 0.83 | 1 (0.8) | 0.85 |
| Max endurance | 84.8 (9.7) | 87.8 (10.6) | 88.1 (9.3) | 3 (11.1) | 1.00 | 3.3 (8.9) | 1.00 |
| 6MWT | 416.4 (16.6) | 407.5 (22.1) | 390.2 (20.8) | -8.9 (16.2) | 0.99 | -26.2 (19.4) | 0.75 |
| SGRQ symptoms | 26.2 (2.7) | 24.6 (4.2) | 33.3 (3.9) | -1.6 (4.6) | 1.00 | 7.1 (4.4) | 0.58 |
| SGRQ activity | 40.8 (2.4) | 37 (4.5) | 39.9 (3.6) | -3.8 (5) | 0.97 | -0.9 (3.9) | 1.00 |
| SGRQ impacts | 18.2 (1.3) | 15.7 (2.7) | 20 (2.7) | -2.5 (2.7) | 0.94 | 1.9 (2.5) | 0.97 |
| SGRQ total | 26.9 (1.6) | 24.3 (2) | 28.6 (1.8) | -2.6 (2.3) | 0.87 | 1.7 (2.2) | 0.97 |
| EQ5DVAS | 68.8 (3.0) | 70.7 (3.6) | 69.8 (3.4) | 1.9 (3.7) | 0.99 | 1 (3.5) | 1.00 |
| EQ5D tot | 0.86 (0.04) | 0.89 (0.04) | 0.89 (0.04) | 0.03 (0.03) | 0.93 | 0.03 (0.04) | 0.99 |
| MRC | 0.83 (0.14) | 0.8 (0.17) | 1.09 (0.23) | -0.04 (0.17) | 1.00 | 0.26 (0.22) | 0.86 |
| CRP | 5.1 (1.0) | 4.6 (1.1) | 7.1 (1.2) | -0.5 (0.7) | 0.98 | 1.9 (0.8) | 0.03 |
| Mg | 0.81 (0.02) | 0.81 (0.02) | 0.82 (0.02) | -0 (0.02) | 1.00 | -0 (0.02) | 1.00 |
| TNF | 5.9 (0.6) | 6.6 (0.6) | 6.6 (0.7) | 0.7 (0.4) | 0.56 | 0.7 (0.4) | 0.59 |

§: with Tukey-Kramer adjustment for multiple comparisons. Abbreviations: FEV1/FVC = Tiffeneau Index; FEV1% = predicted FEV1 (forced expiratory volume in one second); FVC = forced vital capacity; FEV6 = forced expiratory volume in six seconds; PEF = peak expiratory flow; FET100% = forced expiration time at 100% of FVC; Ext peak torque = maximum extension moment; Flx peak torque = maximum flexion moment; Max ext power = maximum extension power; Max flx power = maximum flexion power; Max ext strength = maximum extension strength; Max flx strength = maximum flexion strength; Isom M max = maximum isometric moment; Isom M mean = mean isometric moment; Isom strength = isometric strength; Max handgrip = maximum handgrip strength; Max endurance = maximum handgrip endurance; 6MWT = six-minute walk test; SGRQ = St George Respiratory Questionnaire; EQ5D = EuroQoL 5D; VAS = visual analogue scale; MRC = Modified British Medical Research Council Questionnaire; CRP = C-reactive protein Isom; Mg = magnesium; TNF-α = tumor necrosis factor-α.
