# Supplementary material for: Clinical trial on the effects of oral magnesium supplementation in stable-phase COPD patients
Source: Aging Clin Exp Res. 2021 Jul 14;34(1):167–74. doi: 10.1007/s40520-021-01921-z (PMC8794984; doi:10.1007/s40520-021-01921-z)
Supplement: Supplementary file 2 — Supplementary file2 (DOCX 40 KB) [file 40520_2021_1921_MOESM2_ESM.docx]

**Supplementary Figure 1. Magnesium and COPD study flowchart**

FOLLOW-UP 6 MONTHS

FOLLOW-UP 3 MONTHS

Analyzed (n=25)

Placebo (n=24)

Analyzed (n=24)

Placebo (n=24)

Excluded n=149

-Not interested (n=36)

-Unable to undergo functional tests (n=32)

-FEV1 <30% (n=16)

-Drugs (steroids, insulin, theophylline) (n=15)

-VFG <60 ml/min (n=12)

-Active Cancer (n=9)

-BMI >34.9 kg/m^2^ (n=9)

-Mg taken (n=6)

-FEV >80% (n=5)

-Calcium taken (n=4)

-Hypomagnesemia (n=3)

-Previous adverse events (n=2)

Discontinued treatment n=4

-Personal Problems (n=1)

-COPD riacutization (n=1)

-Hospital admission (n=2)

Discontinued treatment n=0

Discontinued treatment n=6

-Personal Problems (n=2)

-COPD riacutization (n=2)

-Adverse effects (n=1)

-Hospital admission (n=1)

Placebo n=24

Discontinued treatment n=2 Personal Problems (n=2)

Magnesium n=25

ALLOCATION

ENROLMENT

Randomized n=49

Assessed for eligibility

n=198
